# The efficacy of perioperative gabapentin for the treatment of postoperative pain following total knee and hip arthroplasty: a meta-analysis

**DOI:** 10.1186/s13018-020-01849-6

**Published:** 2020-08-15

**Authors:** Jiayu Kang, Zhihu Zhao, Jianwei Lv, Lei Sun, Bin Lu, Benchao Dong, Jianxiong Ma, Xinlong Ma

**Affiliations:** 1 Department of Orthopedics, Jinhua Municipal Central Hospital, Jinhua, Zhejiang Province People’s Republic of China; 2 Tianjin Hospital, Tianjin University, Tianjin, 300211 People’s Republic of China; 3 Biomechanics Labs of Orthopaedics Institute, Tianjin Hospital, Tianjin, People’s Republic of China; 4 Department of Orthopedics, Tianjin Hospital, No. 155, Munan Road, Hexi District, Tianjin City, People’s Republic of China

## Abstract

**Background:**

Postoperative pain after total knee arthroplasty (TKA) and total hip arthroplasty (THA) influence patients’ rehabilitation and life quality. Although gabapentin has been widely used for analgesia, its efficacy is still controversial in TKA and THA. This meta-analysis was performed to assess the efficacy and safety of gabapentin following TKA and THA.

**Method:**

Electronic databases including PubMed, EMBASE, Cochrane Central Register of Controlled Trials, MEDLINE, and ClinicalTrials.gov were comprehensively retrieved for randomized controlled trials from their inception to June 2019. A total of 7 studies, which compared the administration of gabapentin with that of placebo for the treatment of postoperative pain, were included in our meta-analysis. The meta-analysis was performed according to the Preferred Reporting Items for Systematic Reviews and Meta-Analyses (PRISMA) guidelines.

**Result:**

There was no difference in pain score at 24 (*P* = 0.87), 48 (*P* = 0.15), and 72 (*P* = 0.85) h associated with the use of gabapentin. Likewise, no difference in accumulative morphine consumption at 48 h following TKA or THA was found between gabapentin and placebo (DM = − 8.14, 95% CI − 18.55 to 2.28, *P* = 0.13). The incidence of opioid-related adverse effects, including nausea, pruritus, sedation, and dizziness, is no difference between gabapentin and placebo group. However, subgroup analysis indicated that gabapentin could reduce the incidence of pruritus after TKA (RR = 0.35, 95% CI 0.12 to 0.99, *P* = 0.05).

**Conclusion:**

Based on our meta-analysis, gabapentin did not decrease postoperative pain, cumulative morphine consumption, and the incidence of adverse effects after TKA and THA. There was not enough evidence to support the administrations of gabapentin for postoperative pain after TKA and THA.

## Introduction

Total knee arthroplasty (TKA) and total hip arthroplasty (THA) procedures are generally regarded as some of the most successful surgeries and provide significant benefit to patients with severe knee and hip functional impairment [1]. Recently, more than 370 thousand THA and 700 thousand TKA were operated in the USA with an increasing trend in the foreseeable future [2, 3]. However, about half of patients undergoing TKA and THA suffer from severe postoperative pain in spite of multimodal analgesia [4, 5]. Pain after total joint arthroplasty is the most common cause of prolonging length of hospital stay and functional recovery as well as a common reason for readmission [6]. Therefore, the management of pain after total joint arthroplasty has substantial benefits for the patient to achieve early functional recovery.

In recent decades, multimodal analgesia techniques including gabapentin have generally been used for pain control in order to reduce the side effects of morphine such as sedation, nausea, and vomiting [7]. Gabapentin has a molecular structure similar to that of the neurotransmitter γ-aminobutyric acid (GABA) and acts by inhibiting certain calcium channels, which can decrease the release of neurotransmitters so that it has an established role in the treatment of partial seizures as well as neuropathic pain conditions [8]. It has been indicated postoperatively for a variety of surgeries including TKA and THA to reduce postoperative pain and opioid consumption [9].

In the last few years, some randomized controlled trials (RCTs) have been conducted to determine the efficacy of perioperative gabapentin in the management of postoperative pain in patients undergoing TKA or THA [10–12]. However, the opposite conclusions have been reached. Several previous meta-analyses supported the use of gabapentin after THA or TKA, while Hamilton et al. [13] showed that no evidence to support the routine use of gabapentinoids in pain control following TKA. Therefore, the aim of this current meta-analysis was to investigate the effect of the gabapentin in the management of postoperative pain and the risk of drug-related adverse effects following TKA and THA.

## Materials and methods

### Search strategy

This meta-analysis is reported in accordance with the Preferred Reporting Items for Systematic Reviews and Meta-Analyses (PRISMA) statement. Electronic databases including PubMed, EMBASE, Cochrane Central Register of Controlled Trials, MEDLINE, and ClinicalTrials.gov were retrieved for randomized controlled trials (RCTs), cohort studies, and controlled clinical trials (CCTs) from their inception to June 15, 2019. The following medical subject heading terms, keywords, and their combinations were used: “postoperative pain, total hip arthroplasty, total knee arthroplasty, total hip replacement, total knee replacement and gabapentin”. No restrictions were imposed on language or geographic location.

### Inclusion and exclusion criteria

Studies were regarded as eligible for inclusion if they satisfied the following criteria. Population: patients undergoing TKA or THA. Intervention: gabapentin for postoperative pain control. Comparison: placebo or nothing controlled multimodal analgesia method. Outcomes: visual analog scale (VAS) at 24, 48, and 72 h, cumulative morphine consumption (0 to 48 h), and adverse events (sedation, nausea, pruritus, and dizziness). Study design: RCTs, CCTs, and cohort studies.

### Study selection and data extraction

Two independent investigators excluded studies depending on titles and abstracts, and studies that met the inclusion criteria were searched for full-text assessment. Disagreements were resolved by a third reviewer. The following data were extracted from the eligible literature by two investigators independently: first author’s name, publication year, sample size, gabapentin dose and regimen, anesthetic techniques, pain scores, morphine consumption, knee flexion degree, and side effects (nausea, sedation, dizziness, and pruritus). The primary outcome is VAS score with activity at 48 h. Pain at rest was used instead if pain with activity was not reported. The secondary outcome contained VAS score at 24 and 72 h, cumulative morphine consumption at 48 h, and adverse events (sedation, nausea, pruritus, and dizziness). All cumulative morphine consumption was converted to oral morphine equivalent dose.

### Quality assessment

Two reviewers used the criteria outlined in the Cochrane Handbook for Systematic Reviews of Interventions to evaluate the risk of bias in each included study. Disagreements were resolved by consensus. Seven domains related to risk of bias were evaluated in each eligible study as follows: (1) random sequence generation, (2) allocation concealment, (3) blinding of participants and personnel, (4) blinding of outcome assessment, (5) incomplete outcome data, (6) selective reporting, and (7) other bias. The level of the risk of bias was categorized as “low risk,” “high risk,” or “unclear risk.”

### Statistical analysis

Data analysis was performed using the Review Manager Software for Windows (RevMan Version 5.3, Copenhagen; The Nordic Cochrane Center, The Cochrane Collaboration, 2014). For continuous data, the standardized mean difference with 95% confidence interval (95% CI) was calculated. The cumulative morphine consumption was assessed by the mean difference with a 95% CI. Dichotomous data were expressed as the risk ratio indicates the effect of intervention. Statistical heterogeneity of data was evaluated using the *I*^2^ value and chi-squared test. If *I*^2^ > 50% and *P* < 0.05, statistical was considered to be heterogeneous, and the random effects model was used. Otherwise, the fixed effects model was performed for meta-analysis.

## Results

### Search results

A total of 161 relevant studies were retrieved depending on search strategy, and no additional records were found during manual searches of references. Eighty-one studies were removed as duplicate.

**Fig. 1:**
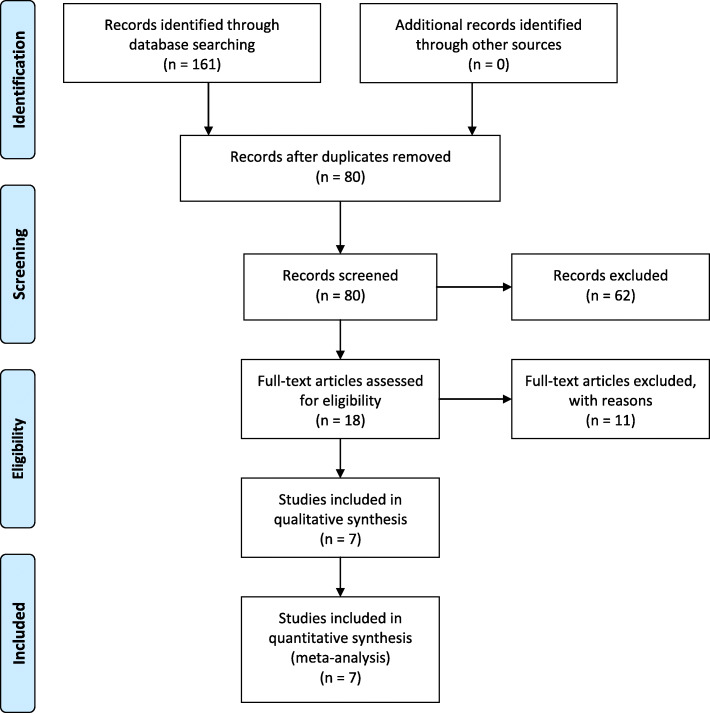
Search result and the selection procedure


After assessing the titles and abstracts of the remaining 80 articles, 62 studies were removed as uncorrelated. Eleven studies were excluded according to the inclusion criteria through reading the full text. During selection, 4 studies were excluded because no subgroup of patients with placebo was analyzed. Seven studies were excluded because patients received a combination of gabapentin and other medicine. Finally, seven randomized controlled trials compared the use of gabapentin after TKA (5 studies) or THA (2 studies) with that of placebo or no treatment (Fig. 1) [14–20]. A total of 837 patients were included in this meta-analysis, and the characteristics are presented in Table 1. Six hundred twenty-one patients of five studies were for TKA, while 216 patients of another two studies were for THA. The 7 trials were published between 2009 and 2019. All 7 studies were RCTs, and one study was retrieved in ClinicalTrials.gov (NCT01680549) [20]. Seven studies compared gabapentin with placebo on postoperative pain according to VAS scale. Six studies reported cumulative morphine consumption at 48 h. Four studies and three analyzed the incidence of drug-related adverse effects (sedation and nausea) respectively.

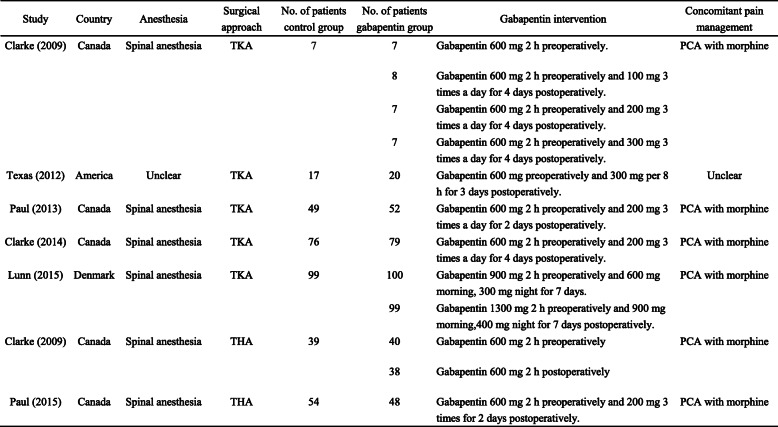

**Table 1:** Description of included studies

Study	Country	Anesthesia	Surgical approach	No. of patients control group	No. of patients gabapentin group	Gabapentin intervention	Concomitant pain management
Clarke (2009)	Canada	Spinal anesthesia	TKA	7	7	Gabapentin 600 mg 2 h preoperatively.	PCA with morphine
					8	Gabapentin 600 mg 2 h preoperatively and 100 mg 3 times a day for 4 days postoperatively.	
					7	Gabapentin 600 mg 2 h preoperatively and 200 mg 3 times a day for 4 days postoperatively.	
					7	Gabapentin 600 mg 2 h preoperatively and 300 mg 3 times a day for 4 days postoperatively.	
Texas (2012)	America	Unclear	TKA	17	20	Gabapentin 600 mg preoperatively and 300 mg per 8 h for 3 days postoperatively.	Unclear
Paul (2013)	Canada	Spinal anesthesia	TKA	49	52	Gabapentin 600 mg 2 h preoperatively and 200 mg 3 times a day for 2 days postoperatively.	PCA with morphine
Clarke (2014)	Canada	Spinal anesthesia	TKA	76	79	Gabapentin 600 mg 2 h preoperatively and 200 mg 3 times a day for 4 days postoperatively.	PCA with morphine
Lunn (2015)	Denmark	Spinal anesthesia	TKA	99	100	Gabapentin 900 mg 2 h preoperatively and 600 mg morning, 300 mg night for 7 days.	PCA with morphine
					99	Gabapentin 1300 mg 2 h preoperatively and 900 mg morning,400 mg night for 7 days postoperatively.	
Clarke (2009)	Canada	Spinal anesthesia	THA	39	40	Gabapentin 600 mg 2 h preoperatively	PCA with morphine
					38	Gabapentin 600 mg 2 h postoperatively	
Paul (2015)	Canada	Spinal anesthesia	THA	54	48	Gabapentin 600 mg 2 h preoperatively and 200 mg 3 times for 2 days postoperatively.	PCA with morphine

### Quality assessment

**Fig. 2:**
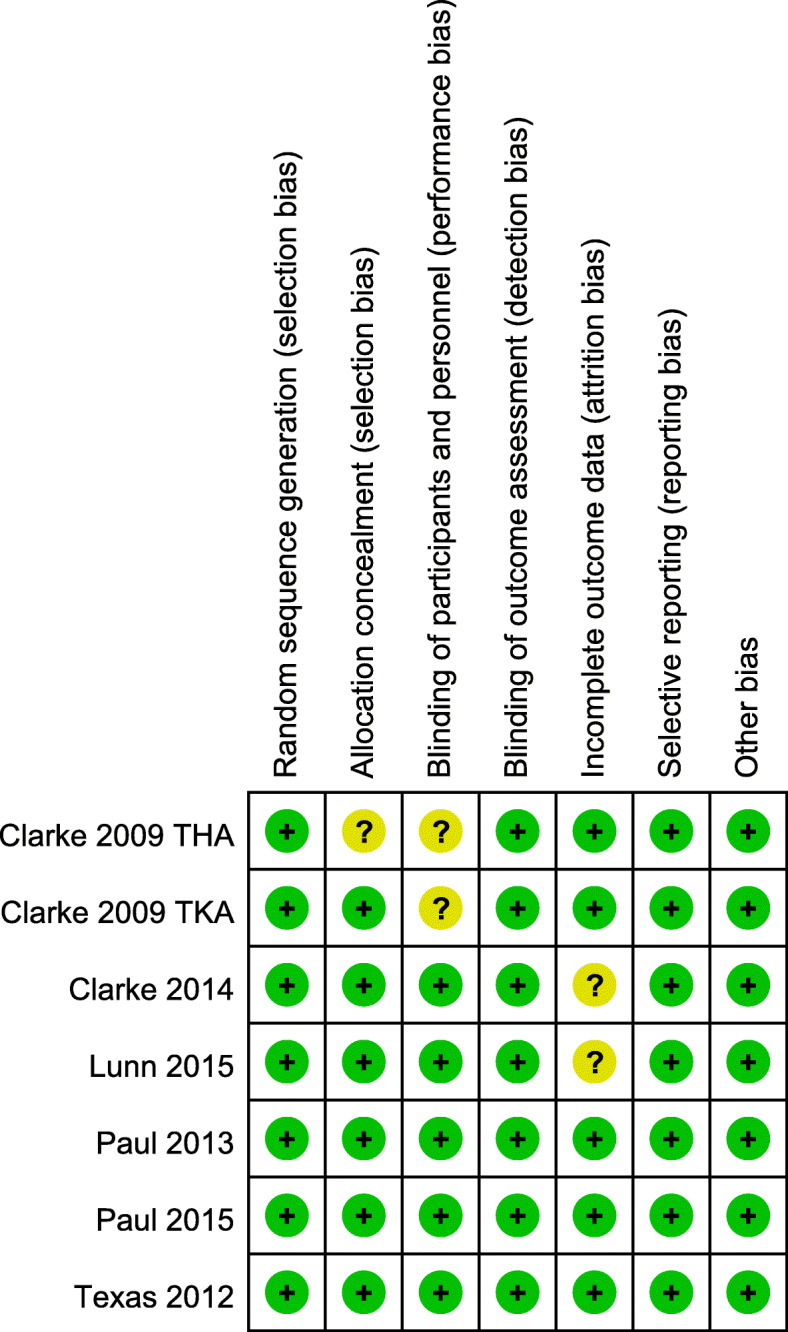
Assessment of risk of bias of included studies

The quality of RCTs is outlined in Fig. 2. Clarke et al. [14] did not describe the details of allocation concealment, blinding of participants and personnel, presenting an unclear risk of bias. Clarke et al. [16] and Clarke et al. [18] did not report the method of blinding of outcome assessment and incomplete outcome data respectively, which presented an unclear risk of bias. A sensitivity analysis was performed according to the risk of bias, and exclusion of studies with high risk of bias did not influence the results.

### Meta-analysis result

#### VAS score at 24 h

**Fig. 3:**
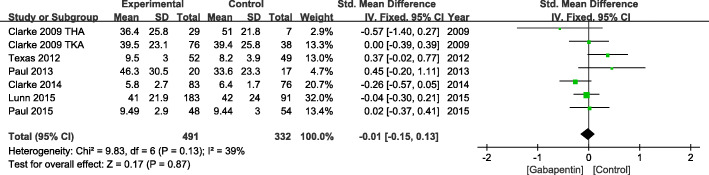
VAS score at 24 h after TKA or THA. VAS, visual analog scale; TKA, total knee arthroplasty; THA, total hip arthroplasty

At 24 h, a total of 7 studies with 823 patients evaluated the VAS score. There was no significant heterogeneity in VAS score at 24 h (*χ*^2^ = 9.83, df = 6, *I*^2^ = 39%, *P* = 0.13) (Fig. 3). Therefore, a fixed effects model was used. Pooled results indicated that compared with placebo, no difference was seen in patients receiving gabapentin (standardized mean difference, − 0.01 [− 0.15, 0.13]; *P* = 0.87) (Fig. 3).

#### VAS score at 48 h

**Fig. 4:**
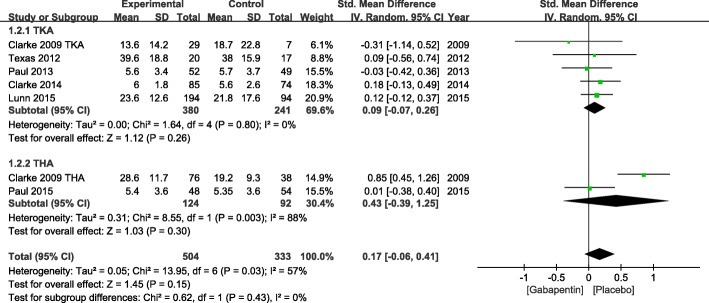
VAS score at 48 h after TKA or THA. VAS, visual analog scale; TKA, total knee arthroplasty; THA, total hip arthroplasty

Data from 7 studies including 837 patients evaluated the postoperative pain regarding the VAS score at 48 h. High heterogeneity limited analysis (*χ*^2^ = 13.95, df = 6, *P* = 0.03, *I*^2^ = 57%). Hence, a random effects model was used, and a subgroup analysis was performed for the VAS score at 48 h. Compared with placebo, gabapentin could not significantly reduce the postoperative pain after TKA and THA at 48 h (SMD = 0.17, 95% CI −0.06 to 0.41, *P* = 0.15) (Fig. 4).

#### VAS score at 72 h

**Fig. 5:**
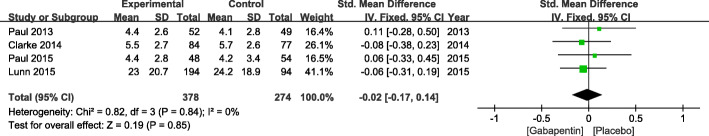
VAS score at 72 h after TKA or THA. VAS, visual analog scale; TKA, total knee arthroplasty; THA, total hip arthroplasty

Only 4 studies with 652 patients reported the postoperative pain according to the VAS score at 72 h. There was no significant heterogeneity (*χ*^2^ = 0.82, df = 3, *P* = 0.84, *I*^2^ = 0%), and a fixed effects model was adopted. No significant difference was found between the gabapentin groups and placebo groups (SMD = − 0.02, 95% CI − 0.17 to 0.14, *P* = 0.85) (Fig. 5).

#### Cumulative morphine consumption

**Fig. 6:**
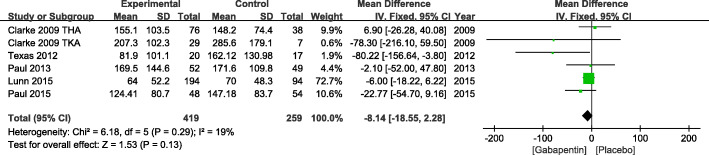
Cumulative morphine consumption (0 to 48 h) after TKA or THA. TKA, total knee arthroplasty; THA, total hip arthroplasty

A total of 678 patients from 6 studies were calculated the cumulative morphine consumption at 48 h. We used a fixed effects model due to the low heterogeneity (*χ*^2^ = 6.18, df = 5, *P* = 0.29, *I*^2^ = 19%). The outcome indicated that gabapentin could not significantly decrease the cumulative morphine consumption at 48 h compared with placebo (DM = − 8.14, 95% CI − 18.55 to 2.28, *P* = 0.13) (Fig. 6).

#### Adverse effects

**Fig. 7:**
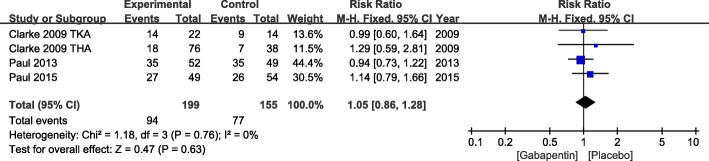
Incidence of sedation after TKA or THA. TKA, total knee arthroplasty; THA, total hip arthroplasty

Four studies reported the incidence rate of sedation. Due to the low heterogeneity (*χ*^2^ = 1.18, df = 3, *P* = 0.76, *I*^2^ = 0%), a fixed effects model was used. No significant increase in the risk of sedation was found in patients who received gabapentin (RR = 1.05, 95% CI 0.85 to 1.28, *P* = 0.63) (Fig. 7).

**Fig. 8:**
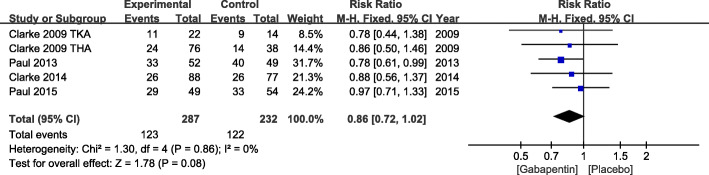
Incidence of nausea after TKA or THA. TKA, total knee arthroplasty; THA, total hip arthroplasty

A total of 287 patients from 5 studies were evaluated the incidence rate of nausea. With no significant heterogeneity, a fixed effects model was adopted (*χ*^2^ = 1.3, df = 4, *P* = 0.86, *I*^2^ = 0%). No significant difference in the incidence of nausea was shown between gabapentin group and placebo (RR = 0.86, 95% CI 0.72 to 1.02, *P* = 0.08) (Fig. 8).

**Fig. 9:**
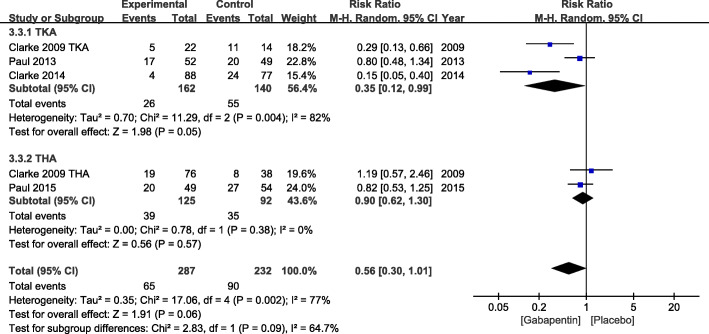
Incidence of pruritus after TKA or THA. TKA, total knee arthroplasty; THA, total hip arthroplasty

The incidence of pruritus was reported in five studies. High heterogeneity influenced the reliability of analysis (*χ*^2^ = 17.06, df = 4, *P* = 0.02, *I*^2^ = 77%). Therefore, a random effects model was used. Although no difference was found in total group (RR = 0.56, 95% CI 0.30 to 1.01, *P* = 0.06), subgroup analysis revealed that gabapentin could reduce the incidence of pruritus following TKA (RR = 0.35, 95% CI 0.12 to 0.99, *P* = 0.05) (Fig. 9).

**Fig. 10:**
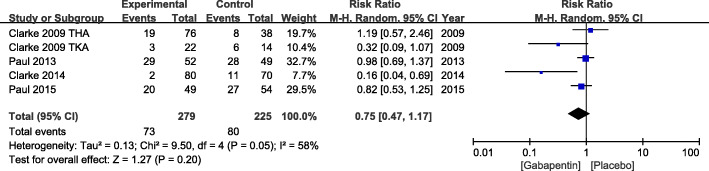
Incidence of dizziness after TKA or THA. TKA, total knee arthroplasty; THA, total hip arthroplasty

Data from five studies including 504 patients assessed the incidence rate of dizziness. With a significant heterogeneity, a random effects model was adopted (*χ*^2^ = 9.5, df = 4, *P* = 0.05, *I*^2^ = 58%). No difference in the incidence of dizziness was detected in gabapentin group (RR = 0.75, 95% CI 0.47 to 1.17, *P* = 0.2) (Fig. 10).

## Discussion

The present meta-analysis was performed to evaluate the effect of gabapentin as a treatment of postoperative pain after TKA or THA. The results indicated that the use of gabapentin is not associated with reducing pain score at 24, 48, and 72 h. There also was no significant difference between gabapentin group and placebo group in the total morphine consumption at 48 h. Compared with control group, gabapentin did not significantly influence the incidence of opioid-related adverse effects, including nausea, sedation, and dizziness. Subgroup analysis indicated that gabapentin is associated with a reduction in the incidence of pruritus (RR = 0.35, 95% CI 0.12 to 0.99, *P* = 0.05) in TKA but no relevance in THA. The pooled results show no evidence to support the routine use of gabapentin in postoperative pain control after total knee arthroplasty or total hip arthroplasty.

The VAS as a common and effective indicator for pain rating was applied to assess the pain level for patients undergoing THA or TKA (0–100 or 0–10, 0 was no pain and 10 or 100 was unbearable pain) [21]. In our meta-analysis, we chose 24, 48, and 72 h as the point-in-time to evaluate the postoperative pain. However, the conclusions of this meta-analysis are contrary to several previous systematic studies evaluating the use of gabapentin for post-surgical pain in several different surgeries, such as cesarean, breast cancer surgery, and spinal surgery [22–25]. The reason for these contradictions may be the different mechanisms and response to pain at different surgical sites [13]. Mishriky et al. [26] reported that there was a significant correlation between types of surgery and postoperative pain scores.

In recent years, some studies share the same views with our meta-analysis on the effect of gabapentin in the management of postoperative pain after surgeries. Erkılıç et al. [27] found that gabapentin reduced IL-6 production on the first 24 h, but the VAS scores at 24 h were not significant difference between gabapentin group and placebo group. No significant difference was found in VAS scores at 12, 24, 48, and 72 h in another meta-analysis [13]. Thus, this meta-analysis indicated that gabapentin cannot significantly reduce the acute pain following TKA or THA.

Although the present study indicated that gabapentin reduced the total morphine consumption by 8.14 mg in the oral morphine equivalent dose at 48 h after TKA or THA, the reduction was not significant. This result of our study is contradictory to previous studies [11, 12]. Possible explanations may be the difference of the surgical procedure and the limitation sample sizes. However, Felder’s [28] meta-analysis shared the same conclusion that no significant differences were found in the use of additional pain medications, supplemental opioids between gabapentin group and placebo group. Subgroup analysis showed that a reduction in the incident rate of postoperative pruritus after TKA was related to gabapentin, but the effect was not found after THA. Furthermore, the risk of other adverse effects including sedation, nausea, or dizziness was not significantly decreased in use of gabapentin, which proved that the reduction in total morphine consumption may not be of clinical relevance.

There are some limitations in our meta-analysis. Only 7 RCTs were included, and the small sample sizes limit the statistical efficacy of our meta-analysis. In addition, variety of study designs and analytical methods may lead to high heterogeneity in included studies. Other factors of included studies including anesthesia methods, way of incision, duration of surgery, and different implants could influence postoperative pain evaluation. Spinal anesthesia was used in 6 of all the 7 studies. It is unclear whether different anesthesia would affect the analgesic effect of gabapentin after TKA or THA. We did not assess TKA or THA postoperative recovery results because of lack of postoperative functional recovery data. Kjaer Petersen et al. [29] reported that the pain or psychological state 3–4 years after TKA are not changed by pre- and perioperative administrations of gabapentin. In our meta-analysis, all included studies did not report long-term follow-up. Furthermore, the dosages and administration time of gabapentin were different in eligible studies. Hu et al. [30] indicated that there was a dose-response relationship in total opioid consumption and postoperative pain for preoperative gabapentin. Therefore, future studies are needed to determine the effect of gabapentin in TKA or THA.

## Conclusion

Based on our meta-analysis, gabapentin did not reduce postoperative VAS scores at 24, 48, and 72 h and postoperative cumulative morphine consumption at 48 h after TKA and THA. Additionally, perioperative administrations of gabapentin did not influence the incidence of opioid-related adverse effects including sedation, pruritus, dizziness, and nausea following TKA or THA. In summary, our meta-analysis indicated that current evidence did not support the routine administrations of gabapentin in postoperative pain control after total hip arthroplasty and total knee arthroplasty. However, further high-quality and large randomized controlled trials are still required to be verified.

## Data Availability

All data and materials were presented in the main paper.

## Abbreviations

CI: Confidence intervals
MD: Mean difference
THA: Total hip arthroplasty
TKA: Total knee arthroplasty
VAS: Visual analog scale
RCTs: Randomized controlled trials
RR: Relative risk
CCTs: Controlled clinical trials
PRISMA: Preferred Reporting Items for Systematic Reviews and Meta-Analyses
